# Magnesium concentration in amniotic fluid in the early weeks of the second trimester of pregnancy

**DOI:** 10.1186/1756-0500-4-185

**Published:** 2011-06-14

**Authors:** Julia Pilar Bocos Terraz, Silvia Izquierdo Álvarez, Jose Luis Bancalero Flores, Ángel González López, Jesús Fernando Escanero Marcén

**Affiliations:** 1 Servicio de Bioquímica Clínica, Hospital Universitario Miguel Servet, Padre Arrupe-Edificio de Consultas Externas (3ª planta), 50009 Zaragoza, Spain; 2 Hospital Militar de Zaragoza-Ministerio de Defensa, Spain; 3 Departamento de Farmacología y Fisiología, Facultad de Medicina, Universidad de Zaragoza, 50009 Zaragoza, Spain

## Abstract

**Background:**

We analyse magnesium levels in amniotic fluid to establish normal values for the 14^th ^to 18^th ^week of pregnancy and establish critical values that could be useful diagnostic and therapeutic guidelines for possible complications.

**Findings:**

Ninety-two samples of amniotic fluid obtained by amniocentesis as well as the corresponding serum samples of pregnant women were analysed. The gestational age (mean ± SD) at which the amniotic fluid sample was obtained was 16.13 ± 1.87 weeks. Magnesium levels were determined by colorimetric assay with chlorophosphonazo-III using the the Cobas c 501 analyser (Roche Diagnostics). Statistical treatment of data was performed using the SPSS program, version 15.0.

Results revealed a mean magnesium value of 1.65 ± 0.16 mg/dL in amniotic fluid and 1.97 ± 0.23 mg/dL in serum.

**Conclusions:**

It would be interesting to extend the study to a larger number of pregnant women to determine variations in normal magnesium values in the three trimesters of pregnancy.

## Background

Amniotic fluid increases in volume as the foetus grows and peaks at an average of 800 mL at approximately 34 weeks of gestation. Approximately 600 mL of amniotic fluid surrounds the baby at full term (40 weeks). This fluid is circulated constantly by the baby inhaling and swallowing existing fluid and replacing it through exhalation and urination [1]. Amniotic fluid accomplishes numerous functions: (1) it protects the foetus from injury by cushioning sudden blows or movements, (2) it enables foetal movement and symmetrical musculoskeletal development, (3) it maintains a relatively constant temperature and thus protects the foetus from heat loss, and (4) it ensures proper foetal lung development [1,2].

Low levels of magnesium in amniotic fluid are associated with pregnancy complications such as preeclampsia [3] and diabetes [4,5]. Magnesium supplements have been demonstrated to reduce the frequency of delayed foetal growth, especially in low-birth-weight babies. However, the general benefits of magnesium for foetal growth and development have not been demonstrated [6]; the Magpie study [7], for example, concluded that treatment with magnesium did not improve preeclampsia.

Consequently, knowledge of the values for this ion in normal amniotic fluid may provide a preventive and early diagnosis of certain maternal and foetal pathologies. Amniocentesis is performed, if indicated, from the second trimester of pregnancy. Although magnesium values in amniotic fluid [8-11] are reported in the literature, no reference values are provided for the three different pregnancy trimesters.

Magnesium in serum is reduced during pregnancy and so additional magnesium is required in the diet [6,12,13]. The recommended daily intake for pregnant women is higher than for the same age group of non-pregnant woman, yet most pregnant women do not consume the required amount [6].

Because variations in magnesium values could be useful as diagnostic and therapeutic guidelines for possible complications, our aim was to determine magnesium levels in amniotic fluid so as to establish normal values for the 14-to-18-week interval of the second trimester of pregnancy.

## Methods

The study design and protocol were reviewed and approved by the "Ethics Committee of Hospital Universitario Miguel Servet" in accordance with the Declaration of Helsinki and the Nuremberg Code. All the patients in the study granted their informed consent.

**Figure 1:**
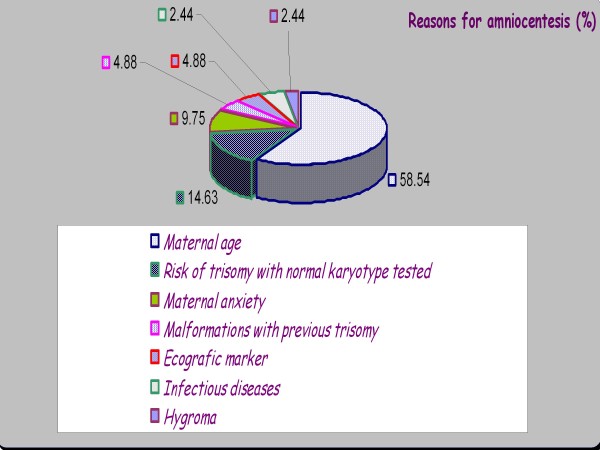
**Reasons for performing amniocentesis in pregnant women**.

Included in the study were 92 pregnant women aged between 21 and 44 years old. Sample size was established on the basis of the mean number of pregnant women undergoing amniocentesis between the 14^th ^and 18^th ^week of pregnancy. The average gestational age at which amniotic fluid samples were obtained by amniocentesis was 16.13 (±1.87) weeks. The extracted amniotic fluid samples were sent immediately to the laboratory for centrifugation and processing to determine magnesium levels, or, if immediate processing was not possible, they were stored at -80°C. The reasons for performing amniocentesis on the patients in our sample are summarized in Figure 1.

Magnesium levels were analysed for both the amniotic fluid and serum of the study population, by colorimetric assay with chlorophosphonazo-III using the Cobas c 501 analyser (Roche Diagnostics). The measurement interval was 0.24-6.08 mg/dL and the lower detection threshold was 0.24 mg/dL. The reference serum value for the normal adult population (21-59 years) was 1.6-2.6 mg/dL. Statistical analysis was performed using the SPSS program, version 15.0.

## Results and discussion

Results revealed a mean amniotic fluid value of 1.65 ± 0.16 mg/dL--with the value 1.6 mg/dL repeated in many samples--and a mean serum value of 1.97 ± 0.23 mg/dL. Amniotic fluid values for magnesium were slightly lower than serum values. No variations in magnesium levels were detected in patients referred for amniocentesis to diagnose disorders that could imply a risk for the foetus. Similarly, despite variations in amniotic fluid volume during pregnancy [1], magnesium concentrations remained fairly stable.

These results are consistent with previous studies [8-11] analysing magnesium levels in amniotic fluid; however, these studies did not distinguish between the three trimesters of pregnancy.

Falls in amniotic fluid magnesium levels are associated with pregnancy complications such as preeclampsia [3] and diabetes [4,5]. Magnesium sulphate, which has a specific maternal central nervous system anticonvulsant action, has gained wide international acceptance as an effective drug for the treatment of eclampsia [14]; however, magnesium treatment does not appear to be effective in preventing preeclampsia. Maternal hypoxia and convulsions, eclampsia and epileptic seizures can have a neurotoxic effect on the foetus. Magnesium sulphate may also protect low-birth-weight infants (under 1500 g) against cerebral palsy [15]. Magnesium is also considered an important factor in the pathogenesis of toxic shock syndrome; according to one study [15], toxin 1 production by *Staphylococcus aureus *was maximal when magnesium concentrations were low, while higher concentrations suppressed toxin 1 production. On the other hand, magnesium sulphate, when given in high dosages to obstetric patients, can cause significant maternal morbidity and rare instances of maternal mortality [14].

## Conclusions

Despite the risk associated with amniocentesis, it would be interesting to extend the study to a larger number of pregnant women and determine magnesium values for the first, second and third trimesters in both normal and abnormal pregnancies, so as to establish normal values and critical ranges and better evaluate complications and their management.

## Competing interests

The authors declare that they have no competing interests. All authors read and approved the final manuscript.

## Authors' contributions

JPBT conceived the study, participated in its design and coordination and helped draft the manuscript. SIA carried out laboratory tests, participated in designing the study and performed the statistical analysis. JLBF participated in the sequence alignment and helped draft the manuscript. AGL carried out the assays and participated in designing the study. JFEM helped draft the manuscript, revised it critically for intellectual content and gave final approval of the version to be published.

## References

[B1] Durlach J Bara M John Libbey Eurotext Le magnésium en biologie et en médicine 2000 221082663

[B2] Natochin IuV Badalian SS Karpenko LA Shakhmatova EI Dynamics of the concentration of copper, potassium, sodium, calcium and magnesium in blood plasma and amniotic fluid in normal and complicated pregnancies Fiziol Cheloveka 1990 16 97 102 2276556

[B3] Dawson EB Evans DR Nosovitch J Third-trimester amniotic fluid metal levels associated with preeclampsia Arch Environ Health 1999 54 412 415 10.1080/00039899909603372 10634230

[B4] Mimouni F Miodovnik M Tsang RC Caliahan J Shaul P Decreased amniotic fluid magnesium concentration in diabetic pregnancy Obstet Gynecol 1987 69 12 14 3796911

[B5] Doraczynska E Rechberger T Wawrzycka B Oleszczuk J Concentration of magnesium in the amniotic fluid of a patient with diabetes Ginekol Pol 1995 66 653 655 8647478

[B6] Yang CY Chiu HF Tsai SS Chang CC Sung FC Magnesium in drinking water and the risk of delivering a child of very low birth weight Magnes Res 2002 15 207 213 12635874

[B7] Magpie Trial Follow-Up Study Collaborative Group The Magpie Trial: a randomised trial comparing magnesium sulphate with placebo for pre-eclampsia. Outcome for children at 18 months BJOG 2007 114 3 289 99 17166221 10.1111/j.1471-0528.2006.01165.x PMC2063969

[B8] Lukácsi L Lintner F Zsolnai B Somogyi J Magnesium transport in human pregnancy (magnesium content of human gestation tissues and tissue fluids) Acta Chir Hung 1991 32 263 268 1842480

[B9] Fabris C Licata D Bertino E Lio C Casolaro F Voglino GF Magnesium concentration in the amniotic fluid Minerva Pediatr 1991 43 3 5 2034192

[B10] Lukácsi L Lintner F Gimes G Zsolnai B Somogyi J Magnesium content of human myometrium and placenta during various stages of gestation, and or different body fluids at term Magnes Res 1993 6 47 52 8369200

[B11] Anastasiadis P Atassi S Rimpler M The concentration of the elements Zn, Cu, Mg, Fe, Na, K in human amniotic fluid during birth J Perinat Med 1981 9 228 234 10.1515/jpme.1981.9.5.228 7288541

[B12] Gortzak-Uzan L Mezad D Smolin A Friger M Huleihel M Hallak M Increasing amniotic fluid magnesium concentrations with stable maternal serum levels: a prospective clinical trial J Reprod Med 2005 50 817 820 16419627

[B13] Chazan B Kowaiska B Pietrasik D Jarosz I Effect of vitamin/mineral supplementation on calcium, magnesium and iron levels in amniotic fluid and serum taken from during first half of pregnancy Ginekol Pol 2001 72 993 996 11883258

[B14] Mittendorf R Pryde PG Elin RJ Cianopoulos JG Lee KS Relationship between hypermagnesaemia in preterm labour and adverse health outcomes in babies Magnes Res 2002 15 253 261 12635881

[B15] Mezad D Hallak M Huleihel M Gortzak-Uzan L Smolin A Mazor M Intravenous magnesium sulphate effect on maternal serum and amniotic fluid cytokines levels in preterm labour patients Magnes Res 2002 15 247 252 12635880

